# Impaired Nuclear Nrf2 Translocation Undermines the Oxidative Stress Response in Friedreich Ataxia

**DOI:** 10.1371/journal.pone.0004253

**Published:** 2009-01-22

**Authors:** Vincent Paupe, Emmanuel P. Dassa, Sergio Goncalves, Françoise Auchère, Maria Lönn, Arne Holmgren, Pierre Rustin

**Affiliations:** 1 Inserm, U676, Hôpital Robert Debré, Bât. Ecran, Paris, France; 2 Faculté de médecine Denis Diderot, IFR02, Université Paris 7, Laboratoire d'Ingénierie des Protéines et Contrôle Métabolique, Paris, France; 3 Département de Biologie des Génomes, Institut Jacques Monod (UMR 7592 CNRS - Universités Paris 6 & 7), Paris, France; 4 Medical Nobel Institute for Biochemistry, Department of Medical Biochemistry and Biophysics Karolinska Institutet, Stockholm, Sweden; Hospital Vall d'Hebron, Spain

## Abstract

**Background:**

Friedreich ataxia originates from a decrease in mitochondrial frataxin, which causes the death of a subset of neurons. The biochemical hallmarks of the disease include low activity of the iron sulfur cluster-containing proteins (ISP) and impairment of antioxidant defense mechanisms that may play a major role in disease progression.

**Methodology/Principal Findings:**

We thus investigated signaling pathways involved in antioxidant defense mechanisms. We showed that cultured fibroblasts from patients with Friedreich ataxia exhibited hypersensitivity to oxidative insults because of an impairment in the Nrf2 signaling pathway, which led to faulty induction of antioxidant enzymes. This impairment originated from previously reported actin remodeling by hydrogen peroxide.

**Conclusions/Significance:**

Thus, the defective machinery for ISP synthesis by causing mitochondrial iron dysmetabolism increases hydrogen peroxide production that accounts for the increased susceptibility to oxidative stress.

## Introduction

Mitochondria are the main site of molecular oxygen utilization in cells. The divalent reduction of oxygen by the respiratory chain is tightly coupled to ATP synthesis by the oxidative phosphorylation machinery [1]. However, monovalent reduction of a small proportion of the available oxygen, which produces superoxide anions, occurs in association with the transfer of electrons through the respiratory chain [2]. During this process, superoxides may be released on both sides of the inner mitochondrial membrane, depending on their production sites [3]. They are subsequently handled by the superoxide dismutases (SODs) present in mitochondria (manganese-dependent SOD in the matrix compartment and copper, zinc-dependent SOD in the intermembrane compartment) [4]. Under basal conditions, progression through the cell cycle is dependent on superoxide production [5]. However, overproduction of superoxides due to abnormal reduction of key components of the respiratory chain (*i.e.*, ubiquinone and cytochrome *b*) or to impairment of antioxidant defenses adversely affects various cellular processes and constituents [2]. Disturbances in either respiratory chain or cellular defenses are being incriminated in an increasing number of acquired and inherited diseases [6]. In this study, we used Friedreich ataxia (FRDA) as a paradigm to investigate the mechanisms involved in impaired cellular responses to mitochondrial oxidative insults. The increased susceptibility to oxidative stress that characterizes FRDA has been documented both *in vitro* [7] and *in vivo* [8], [9], [10]. Progression of the cardiac hypertrophy and of some of the neurological disorders can be slowed by treatment with idebenone, a short-chain ubiquinone homologue with potent antioxidant properties [11], [12].

FRDA is a neurodegenerative condition responsible for cerebellar ataxia and life-threatening cardiomyopathy. Glucose intolerance or diabetes develops in some patients [13]. More than 95% of patients are homozygous for large expansions (60 to 2000) of a GAA triplet–repeat sequence within the first intron of the gene for frataxin, a 210–amino acid protein found in the mitochondrial matrix [14]. The expansion impedes the transcription of the gene and reduces frataxin levels to a few percentage points of normal [15]. Affected tissues are deficient in iron-sulfur cluster (ISC) proteins [16], whose assembly is impaired as a result of inadequate handling of iron [17], [18]. ISC deficiency results in profound deficiencies in the mitochondrial respiratory chain complexes I, II, and III and of the Krebs cycle enzyme aconitase, all of which require ISCs for electron transfer catalysis [16]. Since ISCs synthesized in the mitochondria are subsequently distributed to the various cell compartments [19], the deficiency is not restricted to mitochondrial enzymes but instead affects non-mitochondrial proteins also.

Several tissues (e.g., skeletal muscle) and cells (e.g., skin fibroblasts and circulating lymphocytes) seem able to cope with low frataxin levels and to synthesize enough ISCs to ensure normal production of respiratory chain complexes and aconitase [16]. Nevertheless, fibroblasts from patients with FRDA have impaired responses to oxidative insults, whether endogenous (mitochondrial ATPase blockade by oligomycin) [7] or exogenous (added hydrogen peroxide) [20]. FRDA fibroblasts exhibit actin stress fiber abnormalities [21] and are deficient in glutathione [22]. Previous attempts to determine why these cells are hypersensitive to oxidative stress were unsuccessful. Faulty NF-kB-dependent signaling of antioxidant defenses was ruled out [23]. We recently established that human fibroblasts harboring the ATPase 6 NARP mutation activated the Nrf2-dependent Phase II antioxidant pathway [24]. Under basal conditions, the transcription factor Nrf2 (nuclear factor-erythroid 2-related factor 2) is sequestered in the cytosol, where its Neh2 domain binds to the Kelch domain of the Keap1 protein tethered to actin bundles [25]. These bundles, called actin stress fibers, are found in the center of the cytoplasm and periphery of the nucleus. Cul3-dependent ubiquitination of Nrf2 leads to degradation by the proteasome [26]. When oxidative modification of one of the Keap1 cysteines occurs, Nrf2 escapes from this proteolytic pathway. Phosphorylated Nrf2 then translocates to the nucleus, where it dimerizes with a small Maf protein and binds to the cis-acting antioxidant responsive element (ARE) DNA sequences of Phase II antioxidant genes, activating their transcription [13]. These genes encode the SODs, catalase, glutathione, glutathione reductase (GRed), glutathione-S-transferase (GST), glutamate-cysteine ligase catalytic subunit (GCLC), and NADH quinone oxidoreductase 1 (NQO1) [27], [28].

Here, we investigated the mechanism underlying the increased sensitivity to oxidative stress of frataxin-depleted cells (cultured fibroblasts from patients with FRDA and neuroblastomata-derived SKNAS cells). The Nrf2-dependent signaling pathway was found to be defective. The phenotype associated with the Nrf2-signaling defect was corrected by the catalase mimetic Euk134, emphasizing the key role for the cellular hydrogen peroxide content.

## Results

### Increased sensitivity of FRDA cultured fibroblasts to oxidative stress

**Figure 1:**
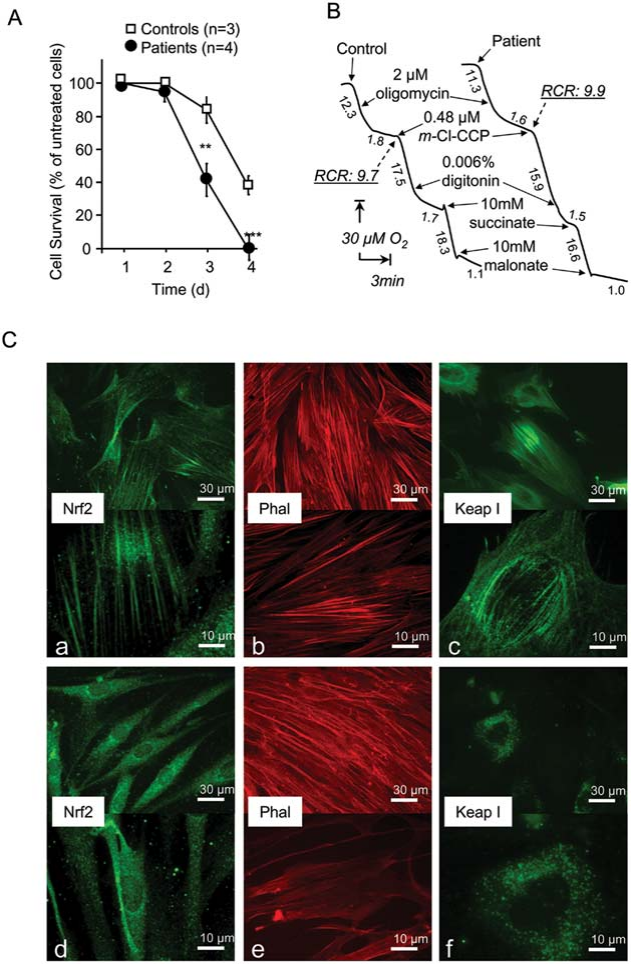
Effect of frataxin depletion on oxidative stress resistance, oxidative properties, and actin-Nrf2 signaling pathway status in cultured skin fibroblasts. A. Sensitivity of control and patient cells to oligomycin (30 µM for 4 days). Cells were harvested at 24-hour intervals and live cells were counted. The proportion of surviving cells was significantly different between controls and patients (ANOVA p<0.01). Significant differences were noted (**p<0.01 and ***p<0.001). Values are means±1 SEM. Open and dark symbols represent control and patient cells, respectively. Filled squares represent patients. B. Effect of frataxin depletion on cell oxidative properties. Under basal conditions, respiration rates were similar between fibroblasts from controls and patients. Adding oligomycin caused greater than 80% inhibition in both cell types. Uncoupled respiration measured in the presence of *m*-Cl-CCP (carbonyl cyanide *m*-chlorophenylhydrazone) decreased progressively when digitonin was added to induce cell permeabilization. Malonate-sensitive mitochondrial succinate oxidation was not different between control and patient cells. Numbers along the traces are nmol/min/mg protein. C. Nrf2/Keap1 localization in patient and control fibroblasts. Labeling of Nrf2, actin (Phal), and Keap1 proteins in control (a, b, c) and patient (e, f, g) fibroblasts, showing disorganization of the actin network and abnormal location of Nrf2 and Keap1 in patient fibroblasts. Experimental procedures are described in the methods section.

SOD levels were moderately but consistently elevated and GSH levels were decreased in severely frataxin-depleted FRDA cultured fibroblasts under basal culture conditions, indicating abnormally high antioxidant activity (Table 1). Cell respiration was normal, and oxidative activity was tightly controlled by the phosphorylation process (Figure 1b). No decreases were found in the activities of the respiratory chain complexes (CI to CV). There was no superoxide overproduction by the respiratory chain, as indicated by the antimycin-resistant cytochrome c reductase activity measured under highly reducing conditions (not shown). Aconitase, an ISC-containing enzyme whose activity is diminished in affected tissues (heart and brain) from FRDA patients, showed similar levels of activity in FRDA and control fibroblasts. Finally, we investigated the glutaredoxin 2 (Grx2) protein, which contains an ISC and protects against oxidative damage by helping to maintain glutathione homeostasis. The ISC stabilizes an inactive form of Grx2, which becomes active upon oxidative disruption of the ISC [29], [30]. We found no significant changes in the Grx2 expression profile between FRDA and control fibroblasts (Supplemental Figure S1 and Supplemental Text S1 for experimental conditions).

**Table 1:** Chronic abnormal redox status in fibroblasts from patients with Friedreich ataxia.

	Frataxin mRNA (% of controls)	Superoxide dismutase activity (IU/mg prot)	Total glutathione (nmol/mg)	GSH/GSSG Ratio
Controls (n = 3)	105.2±6.5 ***	41.1±8.6	94.6±14.8	5.9±1.5 *
Patients (n = 4)	15.1±10.2 ***	61.2±13.1	99.0±23.2	2.4±0.7 *

Experimental conditions as described in the methods section.

* *p*<0.05 and ^***^ *p*<0.001 respectively.

Whereas FRDA fibroblasts proliferate normally under basal conditions, they are oversensitive to the endogenous oxidative insult that results from oligomycin-induced blockade of mitochondrial ATPase [31] (Figure 1A). Similarly, frataxin-depleted cells are oversensitive to endogenous and exogenous oxidative insults [20], [32]. We and others have ascribed this increased sensitivity to impaired induction of antioxidant enzymes in response to oxidative stress [7], [23].

### Abnormal location of the Nrf2 transcription factor in FRDA cultured fibroblasts

Transcription of inducible antioxidants is controlled chiefly by the Nrf2 transcription factor [27], [33]. We found that Nrf2 in control cells was bound to phalloidin-reactive actin stress fibers (Figure 1Ca and b), which also tethered the redox-sensitive Keap-1 protein (Figure 1Cc). In contrast, Nrf2 was not associated with any filamentous structures in FRDA fibroblasts (Figure 1Cd), whose actin stress fibers were abnormally distributed (Figure 1Ce), being found mainly in the cell periphery as previously reported [21]. Finally, Keap1 was not associated with actin stress fibers in FRDA fibroblasts (Figure 1Cf).

### Nrf2 fails to translocate to FRDA cultured fibroblast nuclei in response to oxidative stress

**Figure 2:**
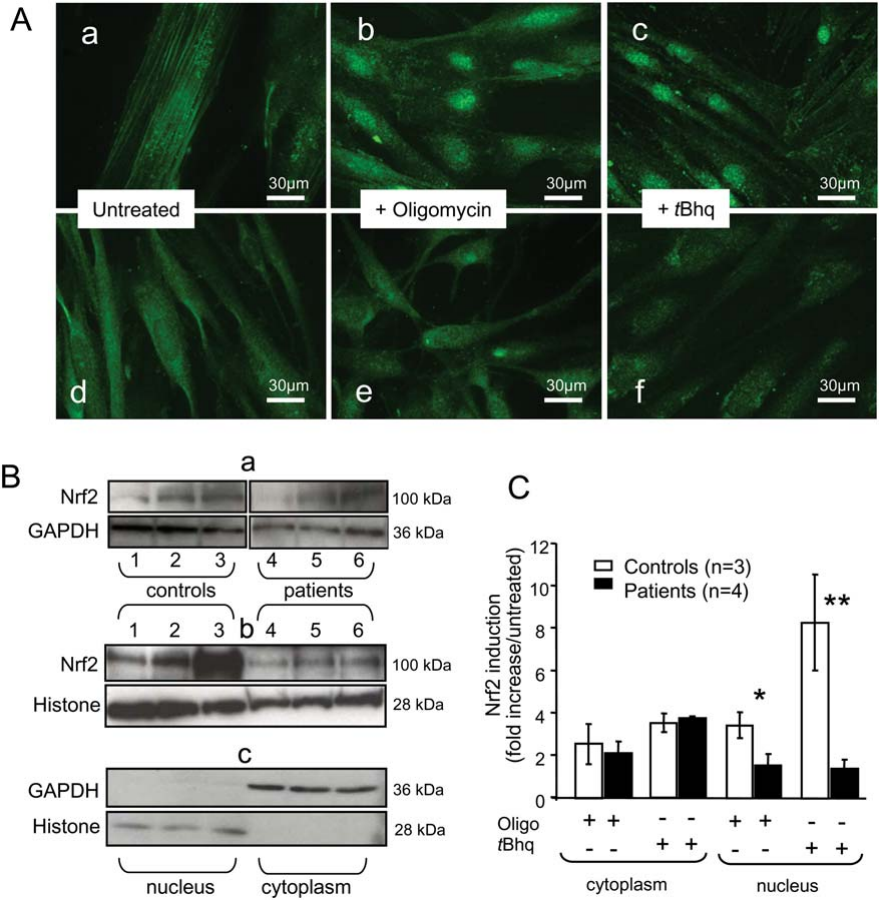
Nrf2 location and amount in control and FRDA patient fibroblasts under basal and oxidative stress conditions. A. Nrf2 localization in control (a, b, c) and patient (d, e, f) fibroblasts under basal conditions (a, d) or after treatment with oligomycin (b, e) or *t*BHQ (c, f). Nuclear Nrf2 translocation occurred in control cells, but not patient cells, after oligomycin or *t*BHQ treatment. B. Western blots of cytoplasmic (a; 40 µg/lane protein) and nuclear (b; 60 µg/lane protein) fractions from control (lanes 1–3) and patient (lanes 4–6) fibroblasts under basal conditions (lane 1, 4), after oligomycin treatment (lane 2, 5), or after *t*BHQ treatment (lane 3, 6). The specificity of the 100 kDa band was confirmed using two antibodies (H-300 and C-20). Fraction purity (d) was assessed by labeling with GAPDH (cytoplasm) and Histone H1 antibody (nuclei). C. Nrf2 content relative to GAPDH and Histone H1 contents in cytoplasmic and nuclear fractions of control and patient fibroblasts. Asterisks denote significant differences (*p<0.05 and **p<0.01). Values are means±1 SEM. Experimental procedures are described in the methods section.

Oligomycin or *t*BHQ was used to induce endogenous or exogenous oxidative stress, respectively. Oligomycin inhibits the enzyme ATPase, thus causing over reduction of the mitochondrial quinone pool, which in turn leads to superoxide overproduction [34]; *t*BHQ undergoes redox cycling either by cellular quinone reductases or via autooxidation reactions, the main end product being hydrogen peroxide [35]. Upon exposure to oligomycin or *t*BHQ, Nrf2 in control cells was released from the actin stress fibers then translocated chiefly to the nuclei (Figure 2A a–c). Nuclear translocation of Nrf2 in FRDA fibroblasts did not occur (Figure 2A d–f). Interestingly enough, neither activation of the Phosphatidyl Inositol 3 kinase (PI3 kinase) by compound 48/80 (2 µg/ml) [36] nor Protein Kinase C (PKC) by phorbol 12-myristate 13-acetate (5 µg/ml) [37] did restore the capacity of a Nrf2 nuclear translocation in FRDA fibroblasts upon an oxidative stress (oligomycin treatment; not shown).

Next, we performed Western blot analyses of nuclei-enriched fractions and cytoplasmic fractions of control and FRDA fibroblasts (Figure 2B). In the cytoplasmic fractions of control and FRDA fibroblasts, Nrf2 content increased 2-fold to 4-fold in response to oligomycin or *t*BHQ (Figure 2B). In the nuclei-enriched fraction of control cells, Nrf2 increased 4-fold in response to oligomycin and 8-fold in response to *t*BHQ. Far smaller Nrf2 increases were seen in the nuclei-enriched fractions of FRDA cells (Figure 2C).

### Decreased expression of Nrf2-targeted genes in FRDA cultured fibroblasts

**Figure 3:**
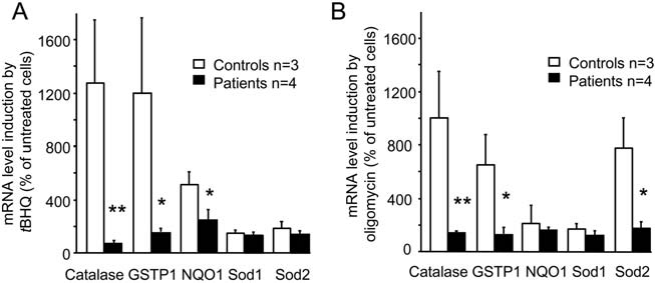
Induction of phase II antioxidants mediated by Nrf2 in control and patient fibroblasts treated with *t*BHQ or oligomycin. Transcription induced by *t*BHQ (A) or oligomycin (B). Significant differences were noted (*p<0.05 and **p<0.01). Values are means±1 SEM. Experimental procedures are described in the methods section.

The failure of Nrf2 to translocate to the nucleus in FRDA cells exposed to oxidative stress prompted us to investigate the expression of genes controlled by Nrf2. In control fibroblasts, *t*BHQ led to greater than 10-fold increases in catalase and glutathione-S-transferase subunit 1 (GSTP1) transcripts, a 5-fold increase in NADH quinone oxidoreductase (NQO1) transcript, and virtually no increases in copper-zinc (Sod1)- or manganese (Sod2)-dependent SOD transcripts (Figure 3A). In FRDA cells, the increases were far smaller; most notably, neither catalase nor GSTP1 transcripts increased noticeably in response to *t*BHQ. Oligomycin treatment of control cells caused large increases in Sod2, catalase, and GSTP1 transcripts; whereas Sod1 and NQO1 transcripts were not substantially increased (Figure 3B). None of these phase II antioxidant enzymes increased in FRDA cells exposed to oligomycin.

### Frataxin-depleted SKNAS cells exhibit the FRDA cell phenotype

**Figure 4:**
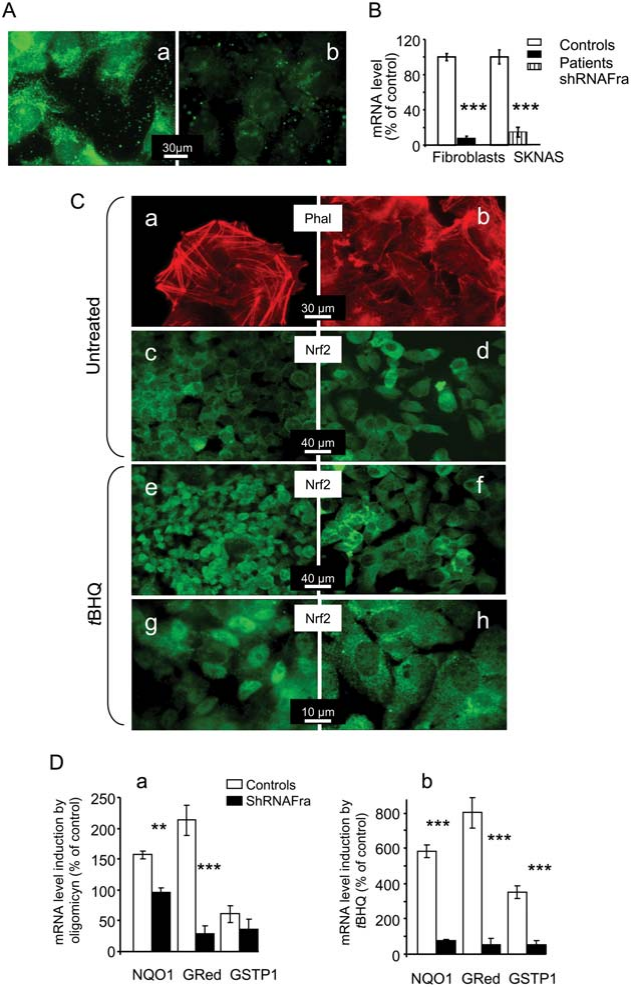
Frataxin-depleted SKNAS cells. A. Anti-frataxin antibody produced strong mitochondrial labeling in control SKNAS cells (a) and severely reduced labeling in SKNAS cells treated with frataxin-targeted shRNA (shRNAFra SKNAS cells) (b). B. Residual frataxin mRNA in patient fibroblasts and shRNAFra SKNAS cells compared to control cells. C. Actin and Nrf2 in control (a, c, e, g) and shRNAFra (b, d, f, h) SKNAS cells under basal conditions (a, b, c, d) or after *t*BHQ treatment (e, f, g, h). Actin staining with phalloidin (Phal) shows disorganization of the actin stress fibers in shRNAFra SKNAS cells (a) compared to control SKNAS cells (b), in keeping with the results in fibroblasts. Nrf2 labeling produced similar abundant staining of the cytoplasm of both control SKNAS cells (c) and shRNAFra SKNAS cells (d). Nuclear translocation of Nrf2 occurs in control SKNAS cells (e, g) but not in shRNAFra SKNAS cells. D. Induction of Phase II antioxidants in SKNAS cells using oligomycin (a) or *t*BHQ (b). Significant differences were noted (**p<0.01 and *** *p*<0.001). Values are means±1 SEM. Experimental procedures are described in the methods section.

The disruption of the Nrf2-signaling pathway in FRDA fibroblasts suggested a mechanism for the hypersensitivity of frataxin-depleted cells to oxidative stress. However, neurons, not fibroblasts, are selectively targeted in Friedreich ataxia. Therefore, we studied neuroblastoma-derived cell lines (SKNAS), using shRNA to silence frataxin. Immunohistochemistry showed severe frataxin-protein depletion in shRNA-treated cells (Figure 4A). The amount of frataxin in these cells was similar to that in FRDA fibroblasts (Figure 4B).

Next, we investigated the organization of actin stress fibers and the location of Nrf2 under basal conditions in SKNAS cells (Figure 4C). Actin stress fibers were visualized by phalloidin labeling in SKNAS cells under basal conditions (Figure 4Ca). Nrf2 labeling, in contrast, did not show the filamentous structures observed in fibroblasts. Frataxin-depleted SKNAS cells exhibited actin network reorganization similar to that observed in fibroblasts (Figure 4Cb). After Nrf2 staining, they were nearly identical to control SKNAS cells (Figure 4Cc and d).

We used *t*BHQ to induce oxidative stress of SKNAS cells. Nuclear translocation of Nrf2 occurred with control SKNAS cells (Figure 4Ce and g) but not frataxin-depleted SKNAS cells (Figure 4Cf and h). Similar results were obtained when control and frataxin-depleted SKNAS cells were treated with oligomycin (not shown). Thus, as with FRDA fibroblasts, frataxin depletion was associated with impairment of the Nrf2 signaling pathway in SKNAS cells.

Finally, we investigated whether the transcription of Nrf2-targeted genes was impaired in frataxin-depleted SKNAS cells exposed to oxidative stress. Treating control SKNAS cells by oligomycin (Figure 4Da) or *t*BHQ (Figure 4Db) resulted in the accumulation of NQO1, glutathione reductase (GRed) and GSTP1, to variable degrees. None of these transcripts increased in frataxin-depleted SKNAS cells exposed to oxidative stress.

### A catalase mimetic restores the Nrf2 response to oxidative stress

**Figure 5:**
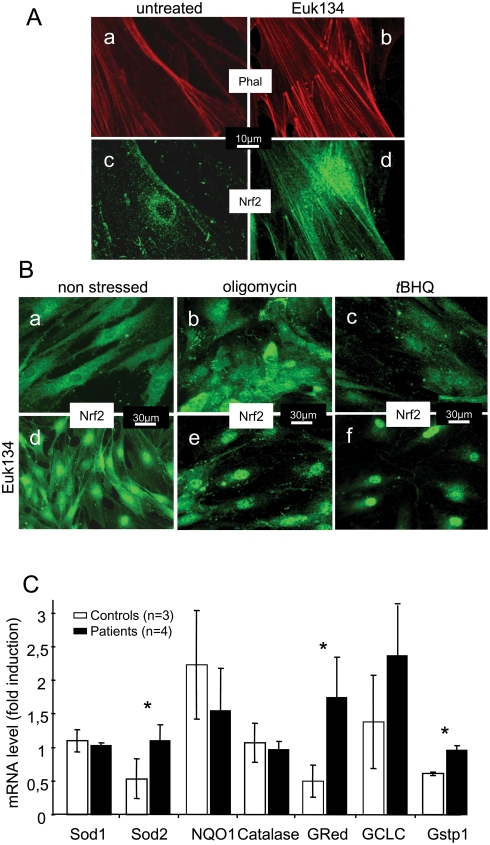
Effect of Euk134 on Nrf2 localization under basal conditions and during oxidative stress in fibroblasts from patients with Friedreich ataxia. A. Staining of actin (a,b) and Nrf2 (c,d) in untreated (a, c) and treated (200 µM Euk134 for 24 h) (b, d) patient fibroblasts. Euk134 restored the normal actin network organization and the location of Nrf2 on the actin bundles. B. Patient fibroblasts under basal conditions (a, d), with oligomycin treatment (b, e), or with *t*BHQ treatment (c, f) after 24 hours pretreatment with 200 µM Euk134 (d, e, f) or without pretreatment (a, b, c). In pre-treated cells, oligomycin and *t*BHQ induced massive nuclear translocation of Nrf2. C. Induction of Phase II antioxidants in control and patient fibroblasts treated with oligomycin. Treatments and experimental procedures are described in the methods section. Significant differences were noted (*p<0.05). Values are means±1 SEM.

Our finding that frataxin-depleted FRDA fibroblasts exhibited a small but consistent increase in peroxide production, in keeping with the higher basal level of SOD (Table 1) led us to investigate the effects of correcting the increased peroxide levels via treatment with the catalase mimetic Euk134. Euk134 is derived from a compound with SOD activity that has been modified to obtain strong catalase activity [38]. Euk134 diffuses freely through the plasma membrane. Euk134 treatment of frataxin-depleted fibroblasts for 24 hours restored the actin stress fibers (Figure 5A a and b) and relocated Nrf2 to the actin filament network (Figure 5A c and d) in a concentration-dependent manner (Table S1). A fraction of the Nrf2 was found in the perinuclear region after Euk134 treatment, resulting in a distinctive perinuclear halo (Fignal 5Ad). These results were not replicated using manganese-tetrakis (4-benzoic acid) porphyrin (MnTBAP) to target superoxides (data not shown) pointing to a specific effect of hydrogen peroxide in the Nrf2 mislocation.

We investigated nuclear Nrf2 translocation in Euk134-treated frataxin-depleted fibroblasts in response to endogenous or exogenous oxidative stress induced by oligomycin or *t*BHQ, respectively. Nrf2 in Euk134-treated fibroblasts was found chiefly in the cytosol under basal conditions (Figure 5Bd). Subjecting these cells to oxidative stress resulted in massive nuclear translocation of Nrf2 (Figure 5Be and f) with marked induction of the Phase II antioxidants Sod2, glutathione reductase, and glutathione-S-transferase (Figure 5C). Thus, the catalase mimetic Euk134 not only relocalized Nrf2 to the actin stress filaments under basal conditions, but also restored the responsiveness of the Nrf2 signaling pathway to superoxides generated by the respiratory chain (oligomycin) or to cytosolic H_2_O_2_ (*t*BHQ). In keeping with this, it has been recently shown that hydrogen peroxide scavenging rescues frataxin deficiency in a Drosophila model of Friedreich's ataxia [39].

Next, we investigated two other approaches designed to restore Nrf2 to its normal location in FRDA fibroblasts. First, we used desferrioxamine or deferiprone for iron chelation (see Supplemental Text for experimental conditions). Both chelators rapidly induced toxic effects, with early aconitase impairment. Neither chelator substantially affected Nrf2 location under our experimental conditions (Supplemental Table S1). Second, to decrease the amount of intracellular peroxides, we provided either reduced glutathione (10 mM) or its precursors N-acetyl cysteine (10 mM) and reduced glutathione ethyl-ester (10 mM). Glutathione has been reported to restore the actin filament network in FRDA cells [21]. However, the actin network was not restored after 48 hours of treatment, whereas Euk134 under similar conditions caused rapid reorganization of the Nrf2 signaling pathway.

## Discussion

Our results shed new light on the response of frataxin-depleted FRDA cells to oxidative stress and provide a rational explanation for the hypersensitivity of these cells to oxidative stress. First, we showed that FRDA cells under basal conditions had a small but consistent elevation in SOD activity and confirmed a reduction in reduced glutathione, indicating that they had to cope with abnormally high levels of superoxides and derived peroxides. These data support the view that frataxin depletion places an increased burden on several antioxidant defense mechanisms under basal conditions. They challenge the recent suggestion [40] that oxidative stress may not be involved in this disease.

Although the increased SOD activity and decreased pool of reduced glutathione suggest increased peroxide production, the antioxidant defense mechanisms of FRDA cells effectively handle this challenge under basal conditions: the cells grow normally, show no increase in apoptotic features, and exhibit normal aconitase activity, which is an exquisitely sensitive marker for oxidative stress [41]. The mechanism by which low frataxin content results in an increased need for antioxidants under basal conditions does not result from a defect in the respiratory chain, which we found normal in frataxin-depleted fibroblasts. Neither has a quantitative abnormality in mitochondrial iron content been demonstrated in patient fibroblasts so far. Qualitative abnormalities are however among the predictable consequences of frataxin depletion with impaired iron chaperoning leading to an increase in iron accessibility and intra mitochondrial iron re location favoring iron reaction with oxygen. Unfortunately, use of iron chelators does not provide a clue to test for oxygen-associated iron toxicity [42]. We showed previously that, although iron chelators protect biological membranes from iron-catalyzed peroxidation (antioxidant effect), they also promote oxidative reactions in the aqueous phase (prooxidant effect), thus merely displacing the target of the oxidative process [43]. However, a role of both oxygen [39], [44] and iron [45], [46] in determining the consequences of frataxin depletion has been demonstrated in a number of models and conditions suggesting that their interaction is actually the critical factor.

Another finding from our study was disorganization of the Nrf2-dependent phase II antioxidant-signaling pathway in FRDA cells under basal conditions. The actin stress fibers were abnormal, as previously reported [21], and we found that the Keap1 and Nrf2 proteins were not in their normal location bound to actin. Induction of phase II antioxidants was absent or severely decreased in response to endogenous and exogenous oxidative stress. Disorganization of the Nrf2 pathway provides an explanation for the impaired induction of antioxidants in response to prooxidant compounds that has been found repeatedly in frataxin-depleted cells. This disorganization was not ascribable to abnormal ATP generation by the respiratory chain, since we found that respiration was not different between FRDA fibroblasts and control fibroblasts. Moreover, the restoration of the actin filament network in FRDA cells treated with the catalase mimetic Euk134 ruled out a role for abnormal ATP generation. We recently reported marked activation of the Nrf2 pathway in fibroblasts exhibiting an ATPase6 mutation responsible for mitochondrial superoxide overproduction by the respiratory chain [24]. A phosphoglycerate mutase (PGAM5) tethers a ternary complex containing Keap1 and Nrf2 to the outer membrane of the mitochondria [47], suggesting that a sub-pool of these proteins might be required to trigger a cell response to mitochondrial oxidative stress.

We found that induction of Phase II antioxidants differed after oligomycin and *t*BHQ exposure. Thus, unknown factors may determine which genes undergo increased transcription mediated by Nrf2.

Frataxin is involved in ISC assembly, thereby regulating mitochondrial iron handling and chaperoning [19]. Our results establish that the Nrf2 pathway plays a crucial role in cells with abnormal mitochondrial iron handling and chaperoning, i.e., frataxin- or Iscu-depleted cells. Interestingly, the hypersensitivity to oxidative stress related to frataxin depletion antedated the development of detectable decreases in iron-sulfur dependent enzyme activities. We carefully investigated the cellular Grx2 content, since the Grx2 ISC may constitute a modular reservoir that might have been affected by a partial and undetected impairment of ISC synthesis. However, Grx2 was not decreased in FRDA cells.

Obviously the existence of feedback-controlled loops between prooxidant and antioxidant components that intimately links oxygen to iron metabolism complicates the identification of the initial step [8]. However, most of the evidence from this study and the literature support a crucial role for hydrogen peroxide. Our finding that a catalase mimetic restored the actin stress fibers and oxidant-induced Nrf2 nuclear translocation in FRDA cells established that hydrogen peroxide was among the early effectors in the toxic cascade of oxidizing reactions. Accordingly, we failed to restore Nrf2 nuclear translocation by activating downstream components of the actin-Nrf2 signaling pathway, such as PI3 kinase or PKC. Similarly, recent results obtained using an FRDA *Drosophila* model support a key role for hydrogen peroxide in the abnormalities associated with frataxin depletion [39]. Finally, the striking analogy between the cerebellar ataxia resulting from either frataxin depletion or alcohol chronic ingestion suggests that a similar H_2_O_2_-dependent actin remodeling hampering anti oxidant defenses in a subset of sensory neurons might well be the actual cause of the progressive cerebellar ataxia rather than the genetically fixed ISP defect.

**Figure 6:**
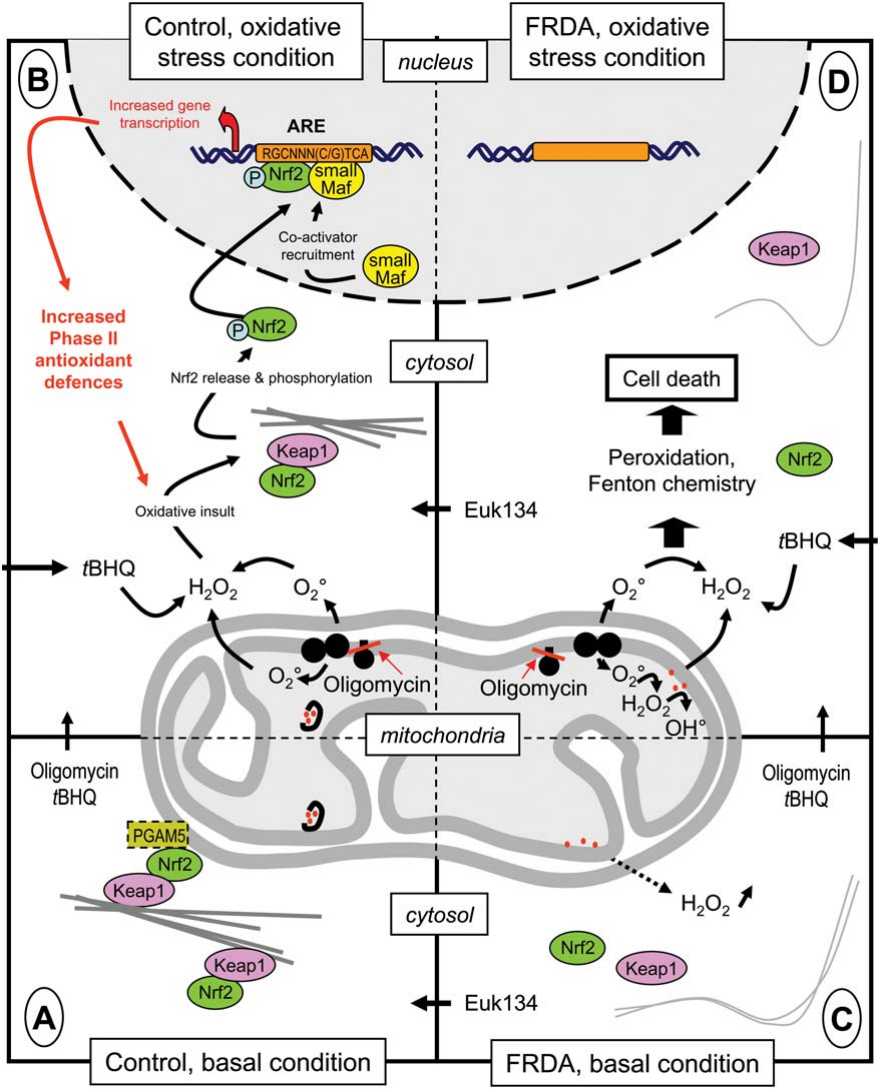
Diagram of the actin-Nrf2 signaling pathway in control and patient cells under basal conditions and during oxidative stress. Under basal conditions, the Nrf2-Keap1 complex, or a sub-pool of it attached to the mitochondrial outer membrane by the PGAM5 protein, is bound to the actin stress filament network of control cells. B. In frataxin-depleted cells, characterized by abnormal iron handling, the actin-Nrf2 signaling pathway is profoundly altered by the need to cope with elevated H_2_O_2_ levels. Removing H_2_O_2_ with the catalase mimetic Euk134 corrects these alterations. C. Treating control cells with oligomycin or *t*BHQ results in major oxidative stress that destabilizes the actin-Nrf2-Keap1 complex, leading to Nrf2 release and phosphorylation. Nuclear translocation of Nrf2 results in the recruitment of the co-activator(s) needed for Phase II antioxidant transcription. D. In frataxin-depleted cells, the oxidative insult induced by oligomycin (endogenous) or *t*BHQ (exogenous) cannot be counterbalanced by the induction of Phase II antioxidants, so that the cells are extremely sensitive to oxidation. Again, Euk134 treatment restores the actin-Nrf2 signaling pathway, allowing transcription of Phase II antioxidants.

In conclusion, our findings suggest an explanation to the hypersensitivity of FRDA cells to oxidative stress, known to be partially counterbalanced *in vivo* by treatment with an antioxidant such as idebenone [12], [48]. Impairment of the Nrf2 signaling pathway leads to failure of antioxidant induction in response to oxidative stress (Figure 6) which suggests methods for restoring the ability of frataxin-depleted cells to respond effectively to oxidative stress. In keeping with this, ligand activation of PPARγ (Peroxisome Proliferator Activated Receptor) by the neuroprotective Pioglitazone, susceptible to increase cell antioxidant defenses [49], [50], may constitute one such method.

## Materials and Methods

### Cell culture

Cultured fibroblasts derived from forearm biopsies were taken from four healthy controls and four patients with FRDA. Patients and controls gave their written informed consent to the biopsy procedure (this study was approved by the Conseil d'Ethique Biomédicale de l'Institut de Recherches Robert Debré/Ir^2^B). In patient fibroblasts, the GAA expansion in the first intron of the FRDA gene was larger than 2.1 kb on the shorter allele. The cells were grown under standard conditions in Dulbecco modified Eagle's medium (DMEM; Gibco, Invitrogen, Cergy Pontoise, France) supplemented with 10% fetal bovine serum, 10 g/ml penicillin/streptomycin, and 2 mM glutamine (Gibco Invitrogen). The medium was changed every three days.

### Cell survival experiment

Cells were seeded in 6-well plates and grown to sub-confluence. The effect on the cells of four days' exposure to 30 µM of oligomycin was tested. The cells were counted after washing with PBS and trypsination, using a Quick Read Precision Cell slide (Globe Scientific Inc., Paramus, NJ).

### Enzyme assays

SOD activity was measured using a Cary 50 UV–visible spectrophotometer (Varian Inc, Les Ulis, France). Activities were determined by monitoring pyrogallol autooxidation at 420 nm and expressed as IU/mg protein. Protein concentrations were measured according to Bradford [51].

### Glutathione measurements

Glutathione levels were determined using a variant of the recycling enzymatic assay [52]. To estimate intracellular glutathione, total proteins were extracted with RIPA buffer. The assay reaction mixture was composed of cell extract, 20 mM DTNB, and 10 mM NADPH in 50 mM potassium phosphate buffer, pH 7.8. The reaction was started by adding glutathione reductase (1.5 units/ml), and the kinetics of DTNB conversion to TNB was followed spectrophotometrically at 412 nm. Glutathione concentrations were calculated from standard curves obtained with various concentrations of reduced glutathione (GSH) and oxidized glutathione (GSSG), based on the rates of TNB formation. For GSSG quantification, samples were pre-treated with 5% (v/v) 2-vinylpyridine for 1 hour at room temperature before analysis.

### Polarography

Respiration of intact cells and oxidation of mitochondrial substrate (using 0.006% digitonin-permeabilized cells) were estimated using polarography, in a magnetically-stirred 250-µl cell thermostated at 37°C (DW1 Clark oxygen electrode; Hansatech Instruments; Norfolk, UK) [53]. All chemicals were of the purest grade available from Sigma–Aldrich (St Quentin; Falavier, France).

### Immunofluorescent staining

Fibroblasts washed with PBS were fixed with 4% paraformaldehyde for 20 min, permeabilized with 0.1% Triton X-100 (w/v) for 5 min, and washed three times with PBS. After blocking with 5% goat serum in PBS/0.05% Tween (v/v) for 30 min, the cells were treated with anti-frataxin antibody, anti-Nrf2 antibody, anti-Keap1 antibody (all diluted 50-fold), or Alexa 568-conjugated phalloidin (diluted 250-fold), for 2 h. The cells were washed three times with PBS/0.05% Tween then treated with anti-rabbit or anti-mouse secondary antibody (diluted 500-fold) for 1 hour. The cells were then washed three times in PBS/0.05% Tween and mounted with fluorescent mounting medium (Dako, Trappes, France). All the procedures were carried out at room temperature. Antibodies to Nrf2 (H300), Keap1 (E-20), and frataxin (H-155) were purchased from Santa Cruz Biotechnology (Heidelberg, Germany). Alexa 568-conjugated phalloidin and Alexa 488-conjugated anti-rabbit secondary antibody were from Invitrogen (Cergy Pontoise, France). Fluorescent microscopy was performed using an LSM5 Exciter microscope (Zeiss; Le Pecq, France) and images were acquired using Axiovision software.

### Treatment with oligomycin, *t*BHQ, and Euk134

Oxidative stress was induced by exposing the cells to oligomycin (30 µM; 18 h) or tertiary-butylhydroquinone (*t*BHQ) (100 µM; 20 h) (Sigma; St Quentin Falavier, France). The cells were then prepared for immunofluorescence staining or harvested for Western blot analysis or total RNA extraction. Oligomycin and *t*BHQ were diluted in ethanol. The final concentration of ethanol never exceeded 0.2%. To test for cellular H_2_O_2_ role in the FRDA cell phenotype, cells were treated with Euk134 (200 µM; 24 h) (Cayman Europe; Tallinn, Estonia) diluted in dimethyl sulfoxide (DMSO), whose final concentration did not exceed 0.1%.

### Western blot experiments

The cytoplasmic and nuclear fractions of fibroblasts were obtained using the NXTRACT kit (Sigma-Aldrich; St Quentin Falavier, France), according to the manufacturer's instructions, except that lauryl-maltosyl (4 mM final concentration) was used as a detergent instead of IGEPAL-CA-630 during cell disruption. The protein concentration was assessed according to Bradford. Samples were heated for 5 min at 95°C with β-mercapto-ethanol, and SDS PAGE Western blotting was performed using standard techniques. Either 40 or 60 µg of protein was loaded on 10% acrylamide gel then transferred to a PVDF membrane (Biorad, Marnes-la-Coquette, France). The membranes were blocked in 5% skim milk in PBS/0.05% Tween and incubated overnight with primary antibodies, at 4°C). Anti-GAPDH (ab8245) and anti-histone H1 (ab11079) antibodies were purchased from Abcam (Paris, France). HRP secondary antibodies (1 h RT) were revealed by ECL (Amersham Pharmacia Biotech, Amersham, UK). Densitometric measures were performed with Image J software.

### Q-PCR experiments

Total RNA was extracted from fibroblasts using the RNeasy®Mini Kit (Qiagen; Courtaboeuf, France) and cDNA was obtained from 1 µg RNA using the RNA PCR Core kit (Roche; Neuilly sur Seine, France) according to the manufacturer's instructions. Quantitative PCR was performed using IQ^tm^SYBR® Green supermix and the MyIQ Single-Color Real-Time PCR Detection System (Biorad). Amplification was achieved using 3 minutes' denaturation at 95°C, 45 denaturation cycles at 96°C for 20 s, annealing at 60°C for 20 s, elongation at 72°C for 20 s, melting at 95°C for 1 min then 55°C for 1 min, and heating to 95°C. RNA was used as the template for the negative-control amplifications included in each PCR run. All reactions were performed in triplicate, and PCR runs were repeated twice. Data were analyzed using MyIQ® software. The amount of target mRNA was normalized for the *HPRT* gene mRNA. Primer pairs were designed to be 20 bp in various exons and to generate products of about 100 bp. Primers were as follows: HPRT, 5′GGTGAAAAGGACCCCACGA3′ and 5′TCAAGGGCATATCCTACAACA3′; NQO1, 5′CATTCTGAAAGGCTGGTTTGA3′and 5′TTGCAGAGAGTACATGGAGC3′; SOD1, 5′TTGGGCAAAGGTGGAAATGAA3′and 5′CACCACAAGCCAAACGACTT3′; SOD2, 5′GGACAAACCTCAGCCCTAACG3′ and 5′TTTGATGGCTTCCAGCAACTC3′; GSTP1, 5′GCAGGAGGGCTCACTCAAA3′ and 5′AGGTGACGCAGGATGGTATT3′; GCLC, 5′TGCTGTCTTGCAGGGAATGT3′ and 5′CACAACCATCCACCACTGC3′; catalase, 5′GTCTGTGTGAGAACATTGCC3′ and 5′ATGTGGCTCCCGTAGTCAG3′; glutathione reductase, 5′ATCCCAACTGTGGTCTTCAG3′ and 5′CACGTTGAATAGGTC TTCACA3′; and frataxin, 5′CCCAGGCTCTCTAGATGAG3′ and 5′GTCCAGTCATAACGCTTAGG3′.

### Short hairpin RNA experiments

Neuroblastoma-derived SKNAS cells were used after frataxin silencing by short hairpin RNA (shRNA). In brief, frataxin-depleted SKNAS cells were obtained by transducing lentiviral particles carrying a gene encoding frataxin-directed shRNA and a puromycin resistance cassette (MISSION™ TRC shRNA; Sigma-Aldrich, St Louis, Missouri). The frataxin-targeting sequence of shRNA was CCGGGCTGGACTCTTTAGCAGAGTTCTCGAGAACTCTGCTAAAGAGTCCAGCTTTTT. SKNAS cells were seeded in 24-well plates. After 24 h, 2 µg/ml hexadimethrine bromide was added just before infection with lentiviral particles. After 18 h, the cells were washed three times with PBS. Successfully infected cells were selected using 10 µg/ml of puromycin (15 d).

### Statistical analysis

Data are reported as means±SEM. Statistical tests were run using Statview software (SAS, Cary, NC). ANOVA was performed, followed by Student's test.

## Supporting Information

Figure S1 Samples were loaded as following: controls (lanes 1, 2 and 3), patients (lane 4, 5 and 6), purified Grx2 (lane 7). α-actin was used as a loading control.
(2.18 MB TIF)

Table S1 Effect of iron chelators and Euk134 on Nrf2 localization in control and patient fibroblasts. Control and patient cells were exposed 24 h either to Deferiprone, Desferoxamine or Euk134. Results reflect percentage of cells showing actin-bound Nrf2.
(0.04 MB DOC)

Text S1 Supplemental Experimental Procedures and figure legend
(0.03 MB DOC)
