# Supplementary figures and images for: Impaired Nuclear Nrf2 Translocation Undermines the Oxidative Stress Response in Friedreich Ataxia
Source: PLoS One. 2009 Jan 22;4(1):e4253. doi: 10.1371/journal.pone.0004253 (PMC2617762; doi:10.1371/journal.pone.0004253)

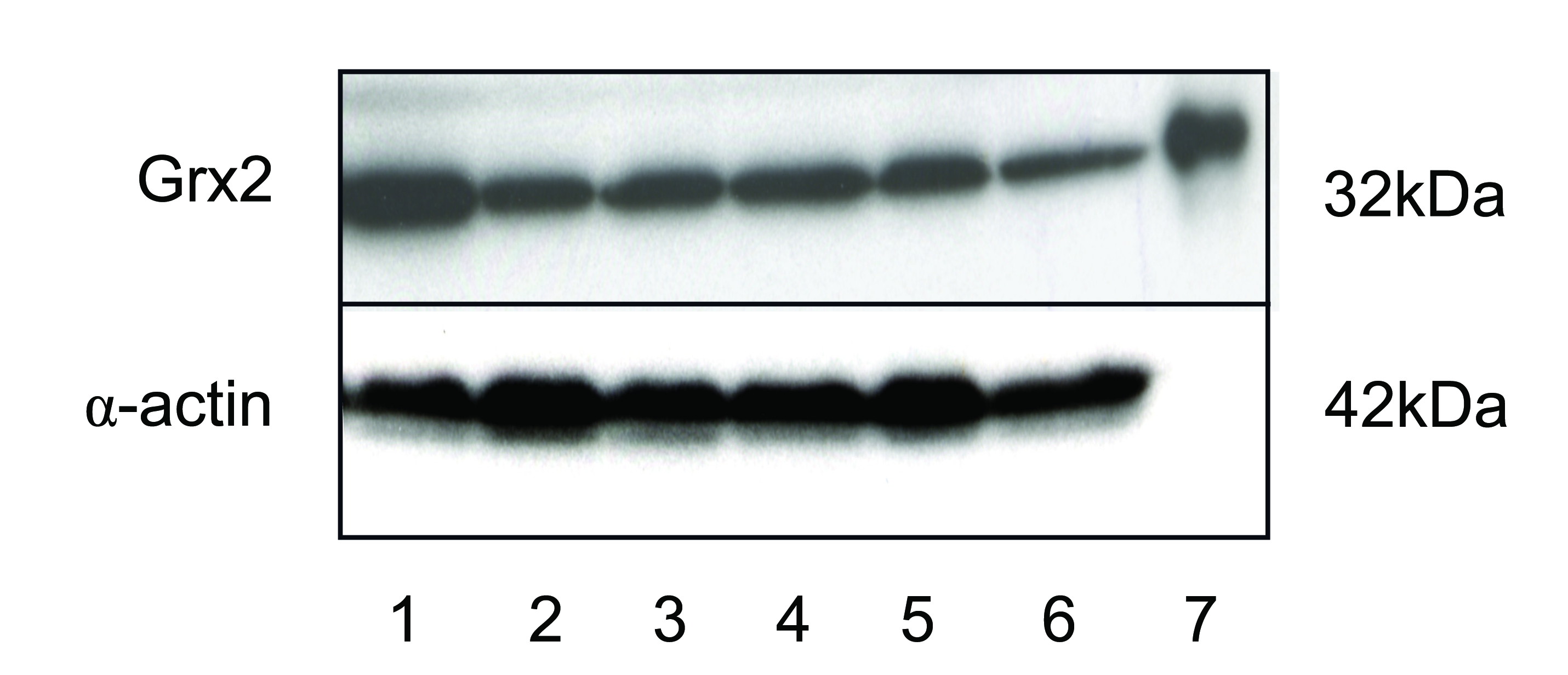

Supplement: Figure S1 — Samples were loaded as following: controls (lanes 1, 2 and 3), patients (lane 4, 5 and 6), purified Grx2 (lane 7). α-actin was used as a loading control. (2.18 MB TIF) [file pone.0004253.s001.tif]
