# Supplementary material for: Impaired Nuclear Nrf2 Translocation Undermines the Oxidative Stress Response in Friedreich Ataxia
Source: PLoS One. 2009 Jan 22;4(1):e4253. doi: 10.1371/journal.pone.0004253 (PMC2617762; doi:10.1371/journal.pone.0004253)
Supplement: Table S1 — Effect of iron chelators and Euk134 on Nrf2 localization in control and patient fibroblasts. Control and patient cells were exposed 24 h either to Deferiprone, Desferoxamine or Euk134. Results reflect percentage of cells showing actin-bound Nrf2. (0.04 MB DOC) [file pone.0004253.s002.doc]

**Supplemental Table**

**Impaired Nrf2 nuclear recruitment undermines oxidative stress response in Friedreich Ataxia**

Vincent Paupe, Emmanuel Dassa, Sergio Goncalves, Françoise Auchère, Maria Lönn, Arne Holmgren and Pierre Rustin

**Table S1**. Effect of iron chelators and Euk134 on Nrf2 localization in control and patient fibroblasts.

Control and patient cells were exposed 24h either to Deferiprone, Desferoxamine or Euk134. Results reflect percentage of cells showing actin-bound Nrf2.

| Desferoxamine | untreated | 50µM | 100µM | 150µM |
| --- | --- | --- | --- | --- |
| Control | 85±7 | 77±10 | 72±11 | 71±9 |
| Patient 1 | 8±5 | 8±5 | 10±7 | 11±5 |
| Patient 2 | 5±5 | 6±5 | 8±6 | 6±5 |
| Patient 3 | 8±5 | 8±7 | 4±5 | 13±6 |
| Patient 4 | 11±5 | 10±5 | 7±5 | 13±4 |

| Deferiprone | untreated | 50µM | 100µM | 150µM |
| --- | --- | --- | --- | --- |
| Control | 85±7 | 77±10 | 72±11 | 71±9 |
| Patient 1 | 8±5 | 5±5 | 7±5 | 5±5 |
| Patient 2 | 5±5 | 6±5 | 8±6 | 5±5 |
| Patient 3 | 8±5 | 5±7 | 4±5 | 13±6 |
| Patient 4 | 11±5 | 7±5 | 5±5 | 17±5 |

| Euk134 | untreated | 100µM | 150µM | 200µM |
| --- | --- | --- | --- | --- |
| Control | 85±7 | 69±7 | 85±7 | 85±7 |
| Patient 1 | 3±6 | 45±13*** | 68±14*** | 66±9*** |
| Patient 2 | 5±5 | 33±17** | 48±7*** | 61±15*** |
| Patient 3 | 9±6 | 28±7** | 51±21*** | 77±14*** |
| Patient 4 | 10±5 | 13±7 | 51±7*** | 55±12*** |

** and *** represent significant difference with untreated condition, values with p<0.01 and p<0.001 respectively.
