# Supplementary material for: Impaired Nuclear Nrf2 Translocation Undermines the Oxidative Stress Response in Friedreich Ataxia
Source: PLoS One. 2009 Jan 22;4(1):e4253. doi: 10.1371/journal.pone.0004253 (PMC2617762; doi:10.1371/journal.pone.0004253)
Supplement: Text S1 — Supplemental Experimental Procedures and figure legend (0.03 MB DOC) [file pone.0004253.s003.doc]

**Supplemental Text**

**Impaired Nrf2 nuclear recruitment undermines oxidative stress response in Friedreich Ataxia**

Vincent Paupe, Emmanuel Dassa, Sergio Goncalves, Françoise Auchère, Maria Lönn, Arne Holmgren and Pierre Rustin

**SUPPLEMENTAL EXPERIMENTAL PROCEDURES**

**Western blot**

Total protein from patient and control cultured skin fibroblasts was extracted with RIPA buffer. Western blots were performed as described previously. Proteins (40 µg) were loaded on a 12% acrylamide gel. Anti-human Grx2 polyclonal antibody was purified from goat serum immunized with recombinant human Grx2. The IgG fraction was obtained by ammonium sulfate precipitation (38% saturation), followed by dialysis against TE buffer (10mM Tris–Cl, 1mM EDTA, pH 7.5).

**Cell treatment**

Cells treated for 24h with the desired compound were washed with PBS twice and immunocytochemistry was performed with anti-Nrf2 antibody as described previously. Nrf2 association to actin bundles was assessed by counting cells showing at least one microfilament labelled with anti-Nrf2 antibody. Deferiprone and Desferoxamine were purchased from Sigma Aldrich (St Quentin Falavier, France) and diluted in water. Euk134 was purchased from Cayman Europe (Tallinn, Estonia).

**SUPPLEMENTAL FIGURE LEGEND**

**Supplementary Figure S1.** Samples were loaded as following: controls (lanes 1, 2 and 3), patients (lane 4, 5 and 6), purified Grx2 (lane 7). α-actin was used as a loading control.
